# Understanding language barriers within patient portals: workarounds and opportunities for Spanish-speaking caregivers

**DOI:** 10.1093/jamiaopen/ooag007

**Published:** 2026-01-27

**Authors:** Gabriel Tse, Stephanie Squires, Jennifer L Carlson, Bonnie Halpern-Felsher, Katherine Hu, Michelle M Kelly

**Affiliations:** Division of Pediatric Hospital Medicine, Department of Pediatrics, Stanford University School of Medicine, Palo Alto, CA 94305, United States; Division of Pediatric Hospital Medicine, Department of Pediatrics, Stanford University School of Medicine, Palo Alto, CA 94305, United States; Division of Adolescent Medicine, Department of Pediatrics, Stanford University School of Medicine, Palo Alto, CA 94305, United States; Division of Adolescent Medicine, Department of Pediatrics, Stanford University School of Medicine, Palo Alto, CA 94305, United States; Division of Pediatric Hospital Medicine, Department of Pediatrics, Stanford University School of Medicine, Palo Alto, CA 94305, United States; Department of Pediatrics, University of Wisconsin School of Medicine and Public Health, Madison, WI 53792, United States

**Keywords:** patient portals, digital health, language equity, machine translation

## Abstract

**Objectives:**

To explore how Spanish-speaking caregivers navigate translation barriers in patient portals and to assess their perspectives on improving language accessibility.

**Materials and methods:**

This qualitative study was conducted at a pediatric academic health system. Semi-structured interviews were conducted with Spanish-speaking caregivers of children with chronic conditions, and inductive thematic analysis was used to generate themes.

**Results:**

Twenty caregivers participated. Three key themes emerged: (1) Caregivers rely on online machine translation tools, which can be inaccurate and time-consuming; (2) Caregivers frequently depend on children and family members for translation, raising concerns about comprehension and appropriateness; (3) Caregivers expressed strong interest in timely and accurate translation features within patient portals to enhance accessibility.

**Discussion:**

Spanish-speaking caregivers develop workarounds to access medical information, but these strategies pose risks to patient safety and exacerbate digital health inequities. While AI-powered machine translation offers a potential solution, concerns about accuracy, regulatory compliance, and equitable implementation must be addressed.

**Conclusion:**

Spanish-speaking caregivers face significant challenges in accessing health information through patient portals. Health systems should prioritize integrated translation solutions, leveraging AI-driven tools while ensuring accuracy and equitable implementation to improve language accessibility.

## Introduction

Patient portals are increasingly used digital tools that allow patients and caregivers to access their medical record through functions such as viewing and making appointments, messaging their providers, and viewing clinical notes. However, significant disparities exist in patient portal access and usage for patients who speak languages other than English (LOE), exacerbating existing health inequities.^1–6^ This “digital divide” has been explained by multiple barriers that include language discordance, limited access to hardware (eg, computers or smartphones) and low digital literacy.^2^^,^^7^^,^^8^

Health systems have attempted to reduce disparities by addressing these barriers, and patient portal vendors are actively developing built-in translation options to improve language concordance.^9^^,^^10^ Despite these advances, Spanish-speaking caregivers continue to experience language-specific barriers, forcing them to develop and employ strategies for overcoming these challenges when navigating their child’s healthcare.^11^ While the experience of Spanish-speaking caregivers accessing pediatric care has been qualitatively investigated, little is known about how Spanish-speaking caregivers navigate translation barriers within patient portals.^11^ Understanding caregiver perspectives is essential for pediatric care delivery, as caregivers are often the primary users of patient portals and the group most affected by language-specific barriers. Gathering these perspectives is particularly timely as artificial intelligence (AI) intelligence technologies are currently being investigated as a tool to translate medical information within patient portals.^12^

Thus, the aim of this study is to explore workarounds that Spanish-speaking caregivers use when faced with language-specific barriers and to elicit their perspectives on how to improve translation within patient portals. Spanish is the most common non-English language in the United States, so understanding strategies that Spanish-speaking caregivers employ to overcome language barriers and incorporating their insights into the development and implementation of AI-driven translation solutions are critical steps toward fostering equitable patient portal access and use.^13^

## Methods

This qualitative study was performed at Stanford Medicine Children’s Health, an academic pediatric health network in Northern California. Our health system uses MyChart (Epic Systems, Verona, WI) as our patient portal platform. We offer both English and Spanish versions of MyChart; while the user interface is translated and patients can send messages in Spanish, unstructured data (eg, clinical notes, reports, medication instructions) is not automatically translated. At our institution, Spanish-speaking patients have a lower patient portal activation rate (55%) compared to English-speaking patients (86%).

We conducted semi-structured interviews with Spanish-speaking caregivers from June to December 2023 with the following inclusion criteria: caregiver has a child <12 years old with at least one chronic condition and has an active patient portal account. Chronic conditions are defined by the Agency for Healthcare Research and Quality as any condition that lasts 12 months or longer and places limitations on self-care, independent living, and social interactions, and/or results in the need for ongoing intervention with medical products, services, and special equipment.^14^ These eligibility criteria were chosen because proxy access changes once a patient turns 12, and patients with chronic conditions often use patient portals more frequently.^15^^,^^16^

Convenience sampling was used to identify participants via electronic health record chart review, based on the availability of the research team. Potential participants were approached and provided written consent in pediatric subspeciality clinics (Allergy, Complex Care, Gastroenterology, Neurology, and Rheumatology clinics). After consent was obtained, a trained, bilingual research team member conducted semi-structured interviews over the phone. We approached 27 potential participants, of whom 20 completed interviews. Among the 7 who did not participate, 3 initially consented but later declined during scheduling, and the remaining 4 could not be reached despite multiple voicemail attempts. Demographic information and reasons for nonparticipation were not available.

The interview guide was developed by the authors as part of a wider study that sought to understand perceived usefulness and facilitators/barriers to using patient portals (Supplement). The Technology Acceptance Model was used as a framework to develop our interview questions.^17^ This was reviewed and piloted with our institution’s Latinx Family Advisory Council and three Spanish-speaking caregivers prior to use. Although the parent study focused on facilitators and barriers to patient portal use, workarounds emerged early in the interviews, and the interviewer added brief probes on this topic as the study progressed. Participants were given a $50 gift card as compensation. COREQ guidelines for the transparent reporting of qualitative research were followed.^18^ Stanford’s Institutional Review Board approved this study.

### Data analysis

Interviews were recorded, transcribed verbatim, and translated into English prior to analysis. Inductive thematic analysis was used as a framework for data analysis.^19^ Two researchers (G.T., S.S.) independently developed codes inductively, then met to adjudicate differences in code and develop a codebook. Codes were organized into categories, which were used to identify themes and subthemes. Three reviewers (B.H.F., J.C., M.M.K.) reviewed and revised the themes and subthemes until consensus was met. Data collection and analysis occurred simultaneously, and interviews were conducted until reaching thematic saturation, which we defined as no new codes arising after two consecutive interviews.^20^ The research team engaged in ongoing reflexive discussion to acknowledge and evaluate how our subjectivity could influence data interpretation.^21^

## Results

Twenty caregivers were interviewed. Participant characteristics are provided in Table 1. Three themes were identified: (1) Caregivers use machine translation software to translate medical information encountered in patient portals; (2) Caregivers rely on their children and other family members to translate medical information found in patient portals; (3) Caregivers express interest in having the ability to automatically translate medical information within patient portals directly (Table 2).

**Table 1.** Participant-reported characteristics, *N *= 20.

Characteristic	*n* (%)
Age, y, mean (SD)	35 (7.4)
Education level	
Less than high school	15 (75)
Completed high school	3 (15)
Some college completed	1 (5)
Completed college or higher	1 (5)
Financial situation	
Does not meet basic expenses	3 (15)
Just meets basic expenses	7 (35)
Meets basic expenses with a little left over	8 (40)
Lives comfortably	1 (5)
Prefer not to say	1 (5)
Ability to speak English	
Very well	0
Well	2 (10)
Not well	9 (45)
Not at all	9 (45)
Ability to read English	
Very well	0
Well	3 (15)
Not well	9 (45)
Not at all	8 (40)

**Table 2.** Themes and representative quotes related to patient portal translation barriers.

Theme	Representative quotes
Use of machine translation software	“I found it difficult because I had to copy and paste, go to Google to translate, and I’d have to then translate it. But as you know, there are many of us parents who are busy, we have a lot of routines, maybe you don’t have enough time or you don’t know how to use this feature, so they find it a bit difficult.”—Participant 14 “I translate them [clinical notes] too. I select it and I click on translate right there, or copy and then I translate it”—Participant 18 “I can translate it with my phone…you just take a picture, let it translate and Google translates it”—Participant 19
Use of children and family members to translate	“Sometimes I do find it complicated doing certain things, and for certain things I do have to ask my daughter to help me, like, to decipher a message or something like that. Because to be honest, I don’t know English all that well.”—Participant 8 “I receive notifications but I tell my daughter, “Come tell me what it says,” and she says, “It’s just a reminder or that they will hold an event or something.” But all of them are necessary, really.”—Participant 13 “Sometimes my daughter translates what they’re saying for me.”—Participant 20
Interest in the automatic translation of text	“Perhaps you can get the feature to change everything in the messages into Spanish, everything into English, everything.”—Participant 11 “The notes…then you just push a button and they get translated into English.”—Participant 9

### Use of machine translation software

Most caregivers (14/20) report using online machine translation software to read and understand text that is not in their preferred language, which can be error prone: *“With that thing that happened about my son’s liver, I put that on the translator, and there were parts it didn’t understand. So I looked for more information, and the word the translator had used was something like my son had something very serious going on with his liver. So I got very worried…[but] it was a translator error”* (Participant 7). Caregivers also describe time consuming processes using translation software: *“I have the (patient portal) messages on my phone…I grab another phone, and with the other phone I take a photo so that the translator translates it for me”* (Participant 8).

### Use of children and family members to translate

Half of caregivers (10/20) reported that they frequently request their children or family members to help translate text within patient portals: *“Sometimes my daughter translates what they’re saying for me.”* (Participant 20). However, caregivers report concern about their children’s ability to accurately translate medical information: *“It’s simply that certain things are not translated, and that there are times when the medications have weird names or things like that, which is hard for my son to translate for me”* (Participant 1).

### Interest in the automatic translation of text

All caregivers (20/20) expressed interest in having the patient portal translated into their preferred language: *“The notes [should be translated], because that’s where you’ll more often find words that you don’t understand, and I think that’s when you could…push a button and they get translated”* (Participant 9). Having timely access to translation text was an important factor, with some caregivers offer a translation button as a solution: *“Maybe it would be important to have the option of translating results. A translation button”* (Participant 11).

## Discussion

Our study reveals that Spanish-speaking caregivers develop workarounds to lack of translation within patient portals, including utilizing machine translation software and asking their children or family members to translate medical information. These workarounds are time consuming, may jeopardize patient safety, and contribute to language inequities within the healthcare system. These findings support prior research that Spanish-speaking caregivers and patients develop various strategies to overcome language barriers.^22^^,^^23^ However, our study uniquely highlights these specific workarounds in the context of patient portals, and reveals caregivers’ interest in having more aspects of the patient portal translated in a timely and safe manner. These results are timely as health systems and patient portal vendors develop and deploy AI-enabled machine translation tools.

Federal regulations support the translation of health information into patients’ preferred languages, though the extent to which this must be implemented remains unclear. Section 1557 of the Affordable Care Act mandates that health systems take reasonable steps to provide meaningful access to health services for patients who speak LOE, and health systems funded by the Department of Health and Human Services are required to provide accurate written translation of “vital documents”.^24^^,^^25^ Translation services must be performed with the assistance of a qualified translator and must not rely on an adult or minor child accompanying the patient.^25^ Despite this, information within patient portals is often not translated, and our study reveals that patients are resorting to workarounds at home that would be unacceptable within health systems.^26^^,^^27^ This further risks exacerbating existing disparities in patient portal access and use among patients who speak LOE, while forcing users to rely on potentially unsafe workarounds.

Spanish-speaking caregivers in our study advocated for a rapid way to translate information within patient portals. While many web-based tools already offer this functionality, unique challenges in clinical settings may hinder adoption. In high-stakes environments such as healthcare, a higher level of accuracy is required to avoid translation errors that could have potential clinical consequences.^28^ From a regulatory standpoint, machine translation tools, including AI applications, are not to be used independently without oversight from a qualified translator.^24^ Prior research examining Google Translate, a widely used machine translation tool, has shown mixed accuracy when translating clinical free text, further underscoring concerns about the reliability of automatic translation software in healthcare settings.^12^^,^^29^^,^^30^

Despite these hurdles, the emergence of large language models presents an opportunity for machine translation tools to rapidly translate clinical text to meet regulatory requirements while promoting language concordance. Preliminary data are promising, showing that some machine translation applications perform comparably to professional translators, and advances in neural techniques have enabled the development of novel multilingual models capable of translating many languages, including low-resource languages.^12^^,^^31–33^ However, disparities in machine translation quality between high- and low-resource languages remain, posing a risk of worsening inequities for patients who speak lower-resource languages.^12^^,^^29^ Future research should evaluate the quality of machine translation across diverse languages and in clinical contexts. Patients should be included in the development and evaluation of translation workflows to ensure that efforts are aligned to patient needs.

### Limitations

Our study interviewed Spanish-speaking caregivers only, and results may not be generalizable to patients who speak other languages. Our participants were already enrolled in patient portals which may represent a greater acceptance of technology compared to the general population. This study was conducted at a single pediatric health network that uses one of multiple commercially available patient portals and may not be generalizable to other health systems that use different patient portal platforms. These findings reflect common patterns raised across a substantial portion of the sample but may not represent the full range of experiences, given that workaround-specific questions were introduced as the study evolved.

## Conclusion

This research highlights that Spanish-speaking caregivers often rely on machine translation software and family members when faced with translation barriers within the patient portal, which can be time consuming and error prone. Health systems should explore novel methods, including machine translation tools, to more rapidly translate health information within patient portals.

## Supplementary Material

ooag007_Supplementary_Data

## Data Availability

The data underlying this article will be shared on reasonable request to the corresponding author.
