# Supplementary material for: Understanding language barriers within patient portals: workarounds and opportunities for Spanish-speaking caregivers
Source: JAMIA Open. 2026 Jan 27;9(1):ooag007. doi: 10.1093/jamiaopen/ooag007 (PMC12870847; doi:10.1093/jamiaopen/ooag007)
Supplement: ooag007_Supplementary_Data [file ooag007_supplementary_data.zip › Supplement Interview Guide.pdf]

INTERVIEW GUIDE:

Thank you for helping with our study. The purpose of this study is to understand your experience of using MyChart so that we can make it better for people who speak Spanish. MyChart is an online tool that allows you to view your child's medical records and speak with your care team. We hope that what we learn from you will help us improve the MyChart experience for families like yours.

Before we start, I want to remind you that we are recording this, but your name will not be attached in any way to the transcript or to any report or publication. Everything we talk about will be confidential. Also, I am not part of the medical team and will not have any influence on your child's medical care. However, if you have a concern you would like to communicate to your medical team, I am happy to help.

This interview is mostly about your own experiences and preferences, and there are no right or wrong answers. I am interested in your honest answers so please feel free to share your thoughts freely.

The interview will last for 60-90 minutes. You will receive a gift card for your participation at the end.

Before we begin, please make sure you are in a quiet location where you feel comfortable talking. *Feel free to open your MyChart account during the interview if you think that will help you answer the questions.* Do you have any questions before we start recording?

Theme 1: Perceived usefulness of MyChart

1. Please tell me about how you use MyChart and what you use it for.

2. Please describe what parts of MyChart you find useful and why they are useful.
    a. E.g., making appointments, attending virtual visits, etc. (prompts only)

3. Have you read your child's notes?
    a. Was it helpful?
    b. How did it affect your understanding of your child's health?

4. What parts of MyChart have impacted your child's health the most? Why?

Theme 2: Facilitators and barriers

5. Is there anything that made it easier for you to use MyChart?

6. Is there anyone that made it easier for you to use MyChart?

   a. When you have issues or need help using MyChart, who do you ask?

7. Please describe what parts of MyChart are difficult to use.

8. What is your favorite part about MyChart? Why?

9. What is your least favorite part about MyChart? Why?

Theme 3: Areas for improvement

10. If you could design a MyChart that was perfect for your needs, what would it look like?
    a. What changes would you recommend making to MyChart?

11. What parts of MyChart would you most want translated into Spanish?
    a. E.g., clinic notes, medications, appointments

12. Would you recommend MyChart to other Spanish-speaking families? Why or why not?
    a. If the hospital CEO came to talk to you about MyChart, what would you say?

13. Can you summarize your opinion of using MyChart in 1-2 sentences?

Final question

14. Do you have any other suggestions or comments about MyChart?
